# EEG Evidence for Dynamic Cross-Modal Adaptation Under Progressive Visual–Tactile Impairment

**DOI:** 10.3390/brainsci16050474

**Published:** 2026-04-28

**Authors:** Hanbo Yang, Yi Wang, Yicheng Sun

**Affiliations:** 1 School of Mechanical Engineering, Xi’an University of Technology, Xi’an 710048, China; yanghanbo@stu.xaut.edu.cn (H.Y.); sunyicheng@stu.xaut.edu.cn (Y.S.); 2 School of Art and Design, Xi’an University of Technology, Xi’an 710054, China

**Keywords:** multisensory integration, cross-modal compensation, visual–tactile interaction, EEG, P300

## Abstract

**Highlights:**

**What are the main findings?**

- Cross-modal compensation in the visual–tactile system showed condition-dependent changes across graded visual and tactile degradation levels, rather than a simple uniform enhancement pattern.
- The compensation effect showed a non-monotonic pattern across conditions, with stronger responses under moderate degradation and weaker responses under more severe dual-modal degradation.

**What are the implications of the main findings?**

- The results are consistent with reliability-dependent changes in sensory weighting under uncertainty, extending current accounts of multisensory integration.
- These findings may inform future work on adaptive human–machine interfaces and safety-critical systems in noisy and degraded environments.

**Abstract:**

Background: This study examined condition-dependent electroencephalography (EEG) changes under progressive degradation of visual and tactile information. Methods: Using a controlled visual–tactile paradigm, we systematically manipulated visual degradation and tactile impairment at multiple levels and analyzed time–frequency activity and P300 responses. Results: The results showed condition-dependent changes in oscillatory activity and P300 amplitude across graded visual–tactile degradation conditions. In several conditions, degradation in one modality was accompanied by increased neural responses in the other modality. However, this pattern was not monotonic: stronger responses were observed under some moderate degradation combinations, whereas responses were reduced under severe dual-modal degradation. Conclusions: In addition, the relative balance between visual-task and tactile-task responses varied across conditions, suggesting flexible but condition-dependent changes in modality weighting rather than a fixed hierarchy between modalities. Overall, these findings are consistent with graded neural adaptation under visual–tactile uncertainty, but they do not by themselves establish a specific causal mechanism of sensory reallocation.

## 1. Introduction

Human perception relies on the coordinated integration of information from multiple sensory channels rather than on any single modality in isolation [1]. Vision, touch, and hearing are continuously combined during perception, decision-making, and action, thereby supporting environmental inference and behavioral stability [2,3,4]. In sensorimotor tasks, the brain must rapidly select, integrate, and update incoming sensory information in order to maintain effective perceptual and behavioral control [5,6,7,8]. Different multisensory combinations have been studied in distinct functional contexts, including audio–visual integration in temporal binding and speech perception [9], auditory–motor coupling in synchronization and timing control [10,11,12,13,14,15,16,17], visual–proprioceptive integration in body representation [18,19,20], and visual–vestibular integration in spatial navigation and balance regulation [21]. For this reason, multisensory integration has become a central topic in research on perception, action, and adaptive behavior.

Among these multisensory combinations, visual–tactile integration is of particular importance because it is closely related to real-world object interaction. Vision primarily provides distal information, such as spatial location, global contour, and expected structure, thereby supporting target localization and anticipatory planning before contact [22]. Touch, by contrast, provides proximal information, including surface texture, local geometric detail, and force-related feedback during contact, and therefore plays a key role in object identification, grasp adjustment, and movement correction [23]. Compared with more extensively studied combinations such as audio–visual integration and auditory–motor synchronization [9,10,11,12,13,14,15,16,17], visual–tactile integration is more directly relevant to object manipulation, grasp control, human–machine interaction, driving-related operations, and other task settings in which perception and action must be coordinated under physical contact constraints. Nevertheless, despite this practical importance, the visual–tactile domain remains less systematically studied, especially under conditions in which the quality of sensory information is degraded.

Most previous studies of multisensory integration have been conducted under conditions in which sensory input is relatively clear and sensory function is assumed to be intact. Under such circumstances, multisensory cues can improve synchronization accuracy and reduce response variability [24,25]. However, real-world environments are rarely optimal. Visual information may deteriorate because of blur, fatigue, or external interference [26,27], whereas tactile information may become less informative because of reduced sensitivity, prolonged contact, or physical barriers between the hand and the object [28,29]. In such situations, the critical issue is not necessarily the absolute loss of a single sensory channel, but rather a change in the relative usability and reliability of information across modalities [30]. Understanding how the brain responds to such graded changes is therefore essential for clarifying how multisensory processing remains adaptive under uncertainty.

Current research in this area still has several important limitations. First, many studies focus on compensation or adaptation after degradation in only one modality, whereas the interactive pattern that emerges when both visual and tactile information are progressively degraded has rarely been examined in a systematic way. Second, sensory degradation is often treated as a binary condition, which makes it difficult to determine whether neural responses change linearly or nonlinearly across degradation levels. Third, much of the available evidence is behavioral, whereas direct electrophysiological descriptions of how neural processing changes across graded visual–tactile degradation conditions remain limited. As a result, it is still unclear whether the relative balance between visual and tactile processing remains fixed, or instead varies as a function of the changing usability of sensory information across conditions.

From a theoretical perspective, when the clarity and usefulness of sensory information vary across modalities, the brain may not maintain a stable sensory hierarchy. Instead, processing may be adjusted according to the relative reliability of the available inputs [30]. In the present context, reliability should be understood as the practical clarity, discriminability, and task relevance of sensory information, rather than as a directly calibrated physical measurement of external signal-to-noise ratio. This perspective provides a useful framework for studying graded multisensory adaptation: by progressively reducing the usability of visual and tactile information, it becomes possible to examine whether neural responses show condition-dependent changes across degradation combinations and whether these changes follow a non-monotonic pattern rather than a simple one-directional increase or decrease.

Electroencephalography (EEG) provides an appropriate method for investigating this issue because of its high temporal resolution. Time–frequency analysis can characterize dynamic changes in oscillatory activity across different frequency bands, whereas the P300 component can provide a useful event-related index of condition-dependent processing [31,32]. Compared with behavioral outcomes alone, EEG measures are more sensitive to subtle differences in neural processing across sensory conditions. Moreover, combining oscillatory measures with P300 amplitude offers a complementary view of how graded visual–tactile degradation may influence neural activity over time. Such an approach is therefore well suited to examining whether progressive dual-modal degradation is associated with systematic and condition-dependent changes in multisensory neural responses.

Accordingly, the present study constructed a graded visual–tactile degradation paradigm in which visual degradation levels and tactile attenuation levels were manipulated separately in order to systematically alter the relative usability of information in the two sensory channels. Using EEG time–frequency analysis together with P300 measures, we aimed to characterize whether neural responses under graded visual–tactile degradation showed condition-dependent patterns, whether these patterns were non-monotonic across degradation combinations, and whether the relative balance between visual-task and tactile-task responses varied across conditions. Rather than directly testing a strong mechanistic model, the present study was intended to provide electrophysiological observations of how neural activity changes under progressive visual–tactile degradation, thereby offering a more concrete basis for understanding multisensory adaptation in complex environments and for informing future work in human–machine interaction, driving safety, and sensory assistance design.

## 2. Materials and Methods

### 2.1. Subject and Environmental Controls

Subject Preparation: Twenty participants were recruited (mean age = 20.62 years), including 10 males. All participants were right-handed, had normal tactile and visual function, and had no history of neurological or psychiatric disorders. Prior to the experiment, participants were instructed to wash their hair prior to EEG preparation to ensure low electrode impedance and ensure that electrode impedance was less than 5 KΩ when wearing the EEG cap. During the experiment, participants were instructed to keep their heads still and minimize movement and ocular artefacts.

Environmental Control: The experiment was conducted in an electromagnetically shielded room, and the environment was kept quiet. Visual stimuli were presented on a screen, while tactile stimuli were delivered by touching physical objects.

### 2.2. Stimulation

Visual Stimuli: Visual stimuli were converted to grayscale and presented under three conditions: no degradation, a 0.3 grayscale-degradation level, and a 0.5 grayscale-degradation level. The 0.3 grayscale-degradation condition was defined as “moderately degraded,” because object contours remained partly recognizable, whereas surface details were substantially obscured, requiring greater cognitive effort for identification. The 0.5 grayscale-degradation condition was defined as “severely degraded,” because visual information became highly degraded and its practical recognizability was markedly reduced.

Tactile Stimulation: Participants performed touch experiments without gloves and while wearing 0.2 mm and 0.5 mm gloves. In the tactile modality, the 0.2 mm glove condition was defined as “mild impairment.” At this level, participants could still perceive the macroscopic geometric shapes of objects (such as spheres or cubes) through active touch, but their ability to distinguish fine surface textures was significantly reduced. The 0.5 mm glove condition was defined as “severe impairment.” At this level, due to the physical barrier of the glove, information regarding the object’s microscopic geometric features and surface textures was completely lost. Participants were unable to reliably identify objects based on tactile input.

In the present study, grayscale degradation and glove thickness were used as graded experimental manipulations intended to reduce the relative clarity and usability of visual and tactile information, respectively. These manipulations should therefore be understood as operational proxies of relative sensory reliability rather than direct physical measurements of sensory signal-to-noise ratio. In other words, the present design was intended to impose graded reductions in the practical usability of sensory information, not to establish quantitatively calibrated sensory SNR levels.

### 2.3. Experimental Procedure

**Figure 1:**
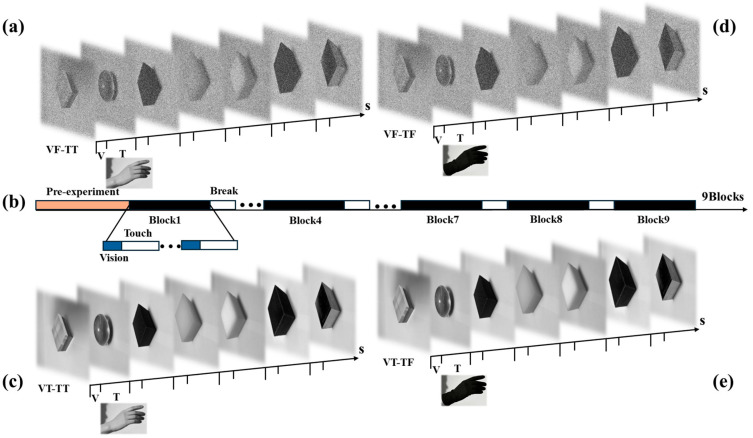
Stimuli and experimental design. (**a**) Severe visual impairment–normal tactile sensation (VF-TT). (**b**) Block design. (**c**) Normal vision–normal tactile sensation (VT-TT). (**d**) Severe visual impairment–severe tactile impairment (VF-TF). (**e**) Normal vision–severe tactile impairment (VT-TF).

This experiment employed a block design (as shown in Figure 1b), with each block consisting of 60 trials and a 5-min rest period between blocks. The experiment comprised a total of 9 blocks, of which Block 1 was Visual Normal–Tactile Normal (VT-TT), as shown in Figure 1c; Block 4 was Severe Visual Impairment–Tactile Normal (VF-TT), as shown in Figure 1a; Block 7 was severe visual impairment–severe tactile impairment (VF-TF), as shown in Figure 1d; Block 8 was normal vision–severe tactile impairment (VT-TF), as shown in Figure 1e. Each trial began with a red fixation point “+” (on a gray background) displayed in the center of the screen. Participants were required to remain seated and fixate on the screen while avoiding blinking and body movements. Data from this phase served as the baseline for subsequent EEG segmentation (−200 ms to 0 ms). Subsequently, a visual stimulus image was displayed on the monitor, depicting one of seven object categories (acrylic block, sphere, granular block, plastic block, rubber block, fuzzy block, or aluminum block). Throughout the experiment, images were presented in a random order, including original grayscale images as well as images presented under the 0.3 and 0.5 grayscale-degradation conditions. At the onset of the visual stimulus, a Trigger 1 signal was sent to the EEG system. The participants were only required to observe; no action was required. After the visual stimulus ended, the participant touched a physical sample of the object to determine whether the object they saw and the one they touched were the same. At the start of the touch (Trigger 2), the system began synchronized recording; subsequently, the participant performed a grasping action, and at the start of the grasp (Trigger 3), the system began synchronized recording. After the grasp was completed, a scoring interface appeared on the screen, asking the participant to subjectively evaluate the fluency of the grasp. After the evaluation was completed, an inter-trial interval began, followed by the automatic start of the next trial.

Figure 1 provides a schematic diagram of representative stimuli and their general task flow, but does not list all the stimuli used in the experiment or all the trial combinations. In the complete experimental arrangement, multiple visual and tactile stimuli are presented throughout the entire experiment, and the visual images presented in each trial do not always correspond one-to-one to the subsequently explored tactile objects. The order of experimental blocks is randomly assigned to each participant rather than being fixed. This helps to reduce systematic effects related to sequence, but still cannot completely eliminate residual learning effects or fatigue effects.

For the EEG analyses reported in the present manuscript, we focused specifically on identity-matched visual–tactile trials. That is, only trials in which the visual stimulus category and the tactile object category were consistent were retained for condition-wise averaging and statistical analysis. Trials with mismatched visual–tactile identities were excluded during preprocessing, because the purpose of the present study was to examine graded changes in relative visual and tactile reliability under matched visual–tactile input, rather than to test congruency conflict effects.

### 2.4. EEG Signal Acquisition

This study utilized the ErgoLAB EEG electroencephalograph, independently developed by China’s KingFar International Inc, for EEG data acquisition. This device uses a sodium chloride solution as the conductive medium, eliminating the need for conductive gel. This not only ensures signal quality but also enhances participant comfort and improves experimental efficiency. The electrodes are arranged according to the international 10–20 system, with a total of 32 channels. The reference electrode employed an average reference method, with the ground electrode positioned at Fz. The data sampling rate was set to 256 Hz, and the online bandpass filter range was set to 1–40 Hz. During the experiment, visual stimuli are presented on a 27-inch high-refresh-rate monitor with a resolution of 1920 × 1080 and a refresh rate of 144 Hz to ensure temporal precision and image smoothness. Concurrently, a second monitor is used to monitor the accuracy of EEG signal acquisition, and a high-definition camera is employed to synchronously record the subject’s behavioral performance.

### 2.5. Data Preprocessing

Data analysis was performed using the Python 3.8 MNE package. The preprocessing workflow included the following steps: ① Raw EEG data were imported into MNE, the electrode coordinate file was loaded, and electrode locations were verified against the international 10–20 system [33,34]. ② The continuous EEG data were band-pass filtered from 0.1 to 30 Hz, using a high-pass cutoff of 0.1 Hz to reduce slow baseline drift and a low-pass cutoff of 30 Hz to attenuate high-frequency noise. In addition, a 50 Hz notch filter was applied to suppress power-line interference [35]. ③ The continuous data were segmented into epochs time-locked to each event of interest (visual stimulus onset, tactile contact onset, and grasp onset), using a time window from −200 to 1000 ms relative to event onset. ④ Baseline correction was applied to each epoch using the mean amplitude during the −200 to 0 ms pre-stimulus interval [36]. ⑤ Artifact rejection was performed in multiple stages. The data were first visually inspected to identify and exclude segments with prominent non-neural artifacts, including excessive slow drift, muscle bursts, and device-related noise. Independent component analysis (ICA) was then applied to the remaining data to identify components reflecting eye blinks, eye movements, cardiac activity, and residual muscle artifacts [37,38]. Components were selected for removal based on their characteristic scalp topographies and time-course features, together with their correspondence to typical ocular, cardiac, or myogenic artifact patterns. After ICA correction, epochs with amplitudes exceeding ±100 μV at any channel were automatically rejected. ⑥ For channels showing persistently poor signal quality throughout the recording, data were reconstructed by spherical spline interpolation from neighboring electrodes. ⑦ Epochs were averaged within each participant and condition to obtain subject-level ERP waveforms and measurements. All group-level visualizations and statistical analyses were then derived from these subject-level estimates, rather than from grand-averaged waveforms alone.

### 2.6. Mathematical and Analytical Framework

To formalize the graded dual-modal degradation paradigm, visual and tactile conditions were represented by normalized degradation parameters D_v_ and D_T_. In the present study, D_v_ ∈ {0,0.3,0.5} and D_T_ ∈ {0,0.2,0.5}, corresponding to intact, moderate/mild, and severe degradation levels in the two sensory channels. Because these values were experimentally assigned condition indices rather than direct physical measurements of external noise variance, they were treated as operational indicators of relative sensory reliability.

Sensory reliability was defined as an inverse function of degradation level:

(1) $$R_{v}=1-D_{v},R_{T}=1-D_{T}$$

where R_V_ and R_T_ denote the relative reliability of visual and tactile inputs, respectively. Based on this formulation, the relative contribution of each modality was interpreted within a reliability-weighted framework:

(2) $$w_{v}=\frac{R_{v}}{R_{v}+R_{T}},w_{T}=\frac{R_{T}}{R_{v}+R_{T}}$$

with

(3) $$w_{v}+w_{T}=1$$

This framework was used as a simplified analytical description of condition-dependent sensory reliability rather than as a forward generative simulation or a direct mechanistic model of neural processing. In other words, it was intended as a conceptual aid for interpreting possible changes in modality weighting across degradation conditions. Hereafter, Vx_Ty is used as a shorthand notation for (D_V_ = x, D_T_ = y).

### 2.7. Time–Frequency Decomposition and Band-Power Extraction

To characterize oscillatory dynamics associated with cross-modal compensation, time–frequency representations were computed in MNE-Python using Morlet wavelet decomposition. For each condition and modality, grand-average evoked responses were loaded and transformed in the frequency range of 1–40 Hz with a frequency resolution of 1 Hz. The number of cycles for each frequency was set to half of the corresponding frequency value (n_cycles = f/2). Inter-trial coherence was not analyzed in the present study.

Baseline correction was applied using the pre-stimulus interval from −200 to 0 ms with log-ratio normalization. To obtain a global representation of oscillatory activity, time–frequency power was averaged across all EEG channels, yielding a global average time–frequency map for each condition. For visualization, a common color scale was used across all panels, defined by the 5th and 95th percentiles of the pooled time–frequency values from all conditions and modalities, in order to facilitate direct cross-condition comparison.

**Figure 2:**
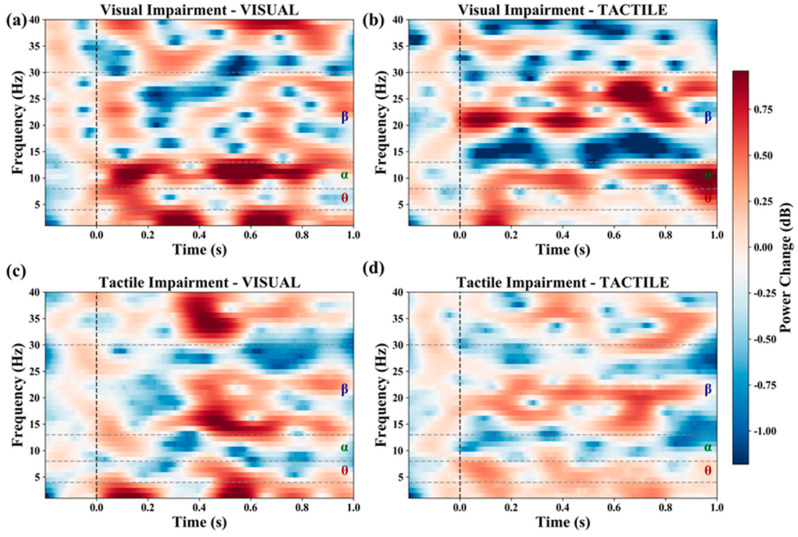
Time–frequency analysis results (global average); (**a**,**b**) represent severe visual impairment with normal tactile perception (V0.5_T0), while (**c**,**d**) represent normal vision with severe tactile impairment (V0_T0.5).

**Figure 3:**
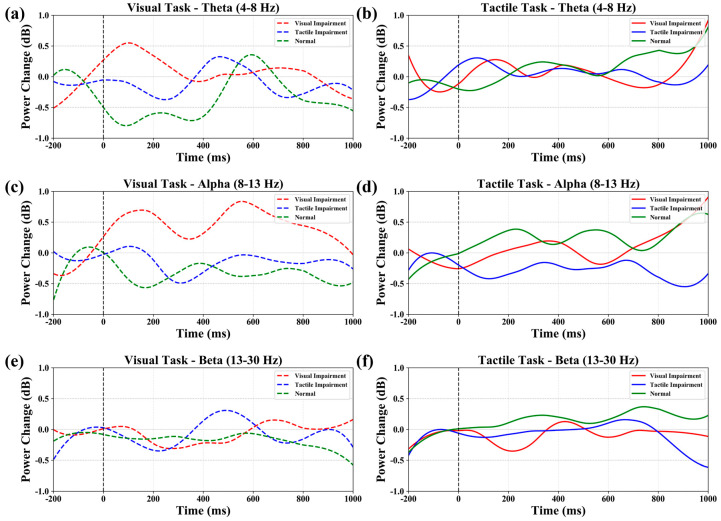
Bandpower plots, where (**a**,**b**) show θ waves during visual and tactile tasks, respectively; (**c**,**d**) show α waves during visual and tactile tasks, respectively; and (**e**,**f**) show β waves during visual and tactile tasks, respectively. The red line represents V0.5_T0, the blue line represents V0_T0.5, and the green line represents V0_T0.

To further quantify the temporal evolution of oscillatory activity shown in the time–frequency maps, band-limited power time series were extracted from the Morlet-based time–frequency representations. Three canonical frequency bands were defined: theta (4–8 Hz), alpha (8–13 Hz), and beta (13–30 Hz). For each condition and modality, frequency bins within the corresponding band were selected, and the baseline-normalized time–frequency power values were averaged first across the selected frequency bins and then across all EEG channels. This procedure yielded a global time-resolved band-power curve for each frequency band. Time was expressed in milliseconds, and the analysis window extended from −200 to 1000 ms relative to stimulus onset. The resulting curves were plotted separately for tactile and visual tasks, with different colors indicating the three representative experimental conditions. The resulting global time–frequency maps under the representative conditions V0.5_T0 and V0_T0.5 were used for the visualization shown in Figure 2, whereas the band-limited power time courses in the theta, alpha, and beta ranges for the visual and tactile tasks were used for the comparisons shown in Figure 3.

### 2.8. P300 Quantification

For each experimental condition, epochs from the same participant and condition were averaged to obtain subject-level event-related potentials (ERPs). P300 quantification was performed at the participant level rather than being read directly from the grand-average waveform. For the visual task, the P300 component was measured at electrode Pz; for the tactile task, it was measured at electrode Cz. In both modalities, the analysis window was defined as 300–500 ms after stimulus onset. Within this window, P300 amplitude was defined as the maximum positive deflection, and peak latency was defined as the time point at which this maximum occurred. Thus, for participant i, visual-task P300 amplitude and latency were defined as

(4) $$A_{i}^{\left(V\right)}=\underset{t\in [300,500]ms}{max}\;ERP_{i}^{\left(V\right)}(Pz,t)$$

(5) $$L_{i}^{\left(V\right)}=arg\underset{t\in [300,500]ms}{max}\;ERP_{i}^{\left(V\right)}(Pz,t)$$

and tactile-task P300 amplitude and latency were defined as

(6) $$A_{i}^{\left(T\right)}=\underset{t\in [300,500]ms}{max}\;ERP_{i}^{\left(T\right)}(Cz,t)$$

(7) $$L_{i}^{\left(T\right)}=arg\underset{t\in [300,500]ms}{max}\;ERP_{i}^{\left(T\right)}(Cz,t)$$

For completeness, mean amplitude within the same window was also extracted during preprocessing, but the statistical analyses and tabulated values reported in the manuscript were based on peak amplitude.

Group-level values reported in Table 1 were obtained by averaging subject-level peak amplitudes across participants. For a given condition, the group mean was calculated as

(8) $$\bar{A}=\frac{1}{n}\sum_{i=1}^{n}\;A_{i}$$

where n is the number of participants and A_i_ is the subject-level P300 peak amplitude for the relevant task. Variability was expressed as the standard error of the mean (SEM), computed as

(9) $$SEM=\frac{SD}{\sqrt{n}}$$

with

(10) $$SD=\sqrt{\frac{1}{n-1}\sum_{i=1}^{n}\;{\left(A_{i}-\bar{A}\right)}^{2}}$$

Accordingly, the values in Table 1 are reported as mean ± SEM for each condition and task modality.

### 2.9. Compensation Metrics and Statistical Analysis

To characterize cross-modal compensation, two complementary indices were computed from the condition-wise group means: absolute increment and compensation percentage. For a comparison between a reference condition and a comparison condition, the absolute increment was defined as

(11) $$\Delta A=A_{comp}-A_{ref}$$

where A_comp_ denotes the mean P300 amplitude in the comparison condition and A_ref_ denotes the mean P300 amplitude in the reference condition. The compensation percentage was calculated as

(12) $$CP(\%)=\frac{A_{\mathrm{comp}\;}-A_{\mathrm{ref}\;}}{A_{\mathrm{ref}\;}}\times 100\%$$

For the visual task (Table 2), compensation was quantified by comparing visual-task P300 amplitudes across tactile impairment levels within the same level of visual impairment. For the tactile task (Table 3), compensation was quantified analogously by comparing tactile-task P300 amplitudes across tactile impairment levels within the same level of visual impairment. These calculations yielded the absolute increment and relative percentage values reported in Table 2 and Table 3.

To compare visual-task and tactile-task P300 amplitudes within each condition, paired-sample *t*-tests were performed on subject-level peak amplitudes. For participant i, the within-condition difference was defined as

D_i_=A_i_^(V)^ − A_i_^(T)^ (13)

The mean paired difference was

(14) $$\bar{D}=\frac{1}{n}\sum_{i=1}^{n}\;D_{i},$$

and its standard deviation was

(15) $$SD_{D}=\sqrt{\frac{1}{n-1}\sum_{i=1}^{n}\;{\left(D_{i}-\bar{D}\right)}^{2}}$$

The paired t-statistic was computed as

(16) $$t=\frac{\bar{D}}{SD_{D}/\sqrt{n}}$$

with degrees of freedom d_f_ = n−1. Effect size was calculated as Cohen’s d for paired differences,

(17) $$d=\frac{\bar{D}}{SD_{D}}$$

The 95% confidence interval (CI) of the mean paired difference was estimated as

(18) $$CI=\bar{D}\pm t_{\alpha /2,n-1}\times \frac{SD_{D}}{\sqrt{n}}$$

**Figure 4:**
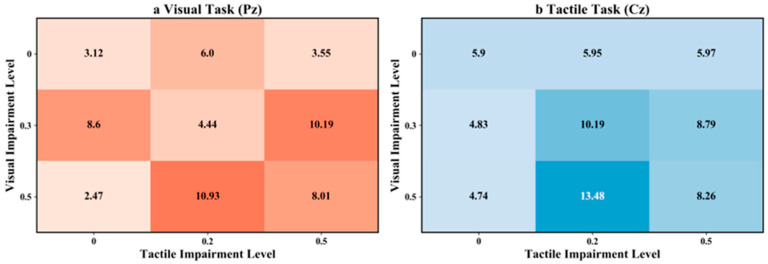
P300 amplitudes under combinations of three levels of visual impairment and three levels of tactile impairment. Here, (**a**) shows P300 values during the visual task, and (**b**) shows P300 values during the tactile task.

**Figure 5:**
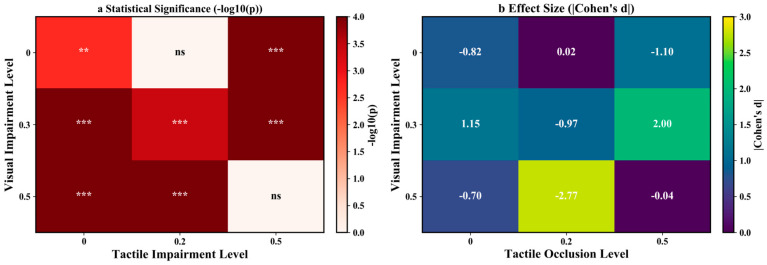
Statistical summary of the comparison between visual-task and tactile-task P300 responses across conditions. (**a**) Heatmap of BH-FDR-adjusted *p* values from paired-sample *t*-tests, where darker colors indicate more significant differences after BH-FDR correction; (**b**) heatmap of Cohen’s d effect sizes. Significance symbols are based on the BH-FDR-adjusted *p* values: *** *p* < 0.001, ** *p* < 0.01, ns = not significant.

To control for multiple comparisons across the nine within-condition paired tests, the resulting *p* values were adjusted using the Benjamini–Hochberg false discovery rate (BH-FDR) procedure. Accordingly, Table 4 reports the exact *p* values, the BH-FDR-adjusted *p* values, Cohen’s d, and the 95% confidence intervals for the paired mean differences. In addition, the condition-wise P300 amplitudes across the 3 × 3 combinations of visual and tactile impairment levels were visualized in Figure 4, whereas the statistical summary of the within-condition visual–tactile comparisons was presented in Figure 5.

## 3. Results

### 3.1. Analysis of the Existence of Cross-Modal Compensation Effects

Under the V0.5_T0 condition, the time–frequency maps showed increased power in the tactile task relative to the visual task, particularly in higher-frequency ranges (Figure 2b). By contrast, under the V0_T0.5 condition, the tactile task showed reduced power in parts of the time–frequency map, whereas the visual task showed relatively stronger activity in the theta and beta ranges (Figure 2c,d). Taken together, these observations indicate that degradation in one modality was accompanied by condition-dependent changes in oscillatory activity in the other modality. The present results do not by themselves establish a specific neural allocation mechanism, but they are consistent with shifts in relative task-related neural engagement across conditions.

### 3.2. Nonlinear Characteristics of Cross-Modal Compensation

To further quantify the frequency-specific nature of cross-sensory compensation, we conducted a quantitative comparison of power in the θ, α, and β bands under three conditions: severe visual impairment with normal tactile sensation (V0.5_T0); normal vision with severe tactile impairment (V0_T0.5); and normal vision with normal tactile sensation (V0_T0).

Across the three representative conditions, oscillatory activity showed clear condition-dependent modulation in the theta, alpha, and beta bands. Under V0.5_T0, visual-task theta activity was enhanced in the early phase and reduced later, whereas tactile-task enhancement was more evident in the later period. Under V0_T0.5, the visual task showed relatively stronger mid-phase theta activity, whereas the tactile task showed weaker late beta activity. By comparison, the V0_T0 condition showed comparatively stable band-power dynamics across time. Overall, these patterns indicate that asymmetric sensory degradation was associated with condition-dependent differences in oscillatory activity across tasks and time windows, whereas the intact condition produced more stable responses.

### 3.3. Analysis of P300 Amplitude

To further systematically elucidate the dynamic patterns of this compensatory effect as a function of the degree of sensory impairment, we conducted a comprehensive analysis of P300 amplitudes across nine experimental conditions—comprising three levels of visual impairment and three levels of tactile impairment (Figure 4 and Table 1).

Following the recommendations in the literature [31,32], the parietal electrode (Pz) was selected for P300 analysis of the visual task, while the central electrode (Cz) was selected for P300 analysis of the tactile task. Table 1 lists the P300 amplitudes for each condition. As shown in the baseline condition (V0_T0), the P300 amplitude for the visual task was 3.12 ± 0.82 μV, while that for the tactile task was 5.90 ± 0.72 μV. A significant difference was observed between the two, reflecting the classic modality-specific distribution pattern.

Under severe visual impairment (V0.5), the largest visual-task and tactile-task P300 amplitudes were observed at T0.2 (10.93 μV and 13.48 μV, respectively). Under T0.5, both values were lower (approximately 8.1 μV), indicating that the responses were reduced under the most severe dual-modal degradation combination relative to V0.5_T0.2.

### 3.4. Cross-Modal Compensation Effects in the P300 for Visual Tasks

To quantify the effects of tactile degradation on visual-task P300 under different levels of visual degradation, we calculated the relative percentage change and absolute increment for each comparison (Table 2). Two patterns were observed. Under moderate visual impairment (V0.3), mild tactile impairment (T0.2) was associated with a lower visual-task P300 than T0, whereas severe tactile impairment (T0.5) was associated with a higher visual-task P300 than T0. Under severe visual impairment (V0.5), both tactile impairment levels were associated with higher visual-task P300 amplitudes than T0, with the larger increase observed at T0.2. These results indicate that the visual-task P300 did not vary monotonically across tactile impairment levels.

In addition, under V0.5_T0.2, the absolute increase in visual-task P300 was 8.46 μV (Table 2), which was similar in magnitude to the corresponding increase observed in the tactile task (8.74 μV; Table 3). This pattern suggests that tactile degradation was associated with enhanced visual-task responses under some degraded visual conditions. However, the present data do not directly identify the underlying cortical mechanism.

### 3.5. Cross-Modal Compensation Effects of the P300 in Tactile Tasks

The tactile-task P300 also showed a condition-dependent pattern across visual and tactile degradation combinations (Table 3). Under intact vision (V0), tactile-task P300 amplitudes changed only minimally across tactile impairment levels. Under visual impairment (V0.3 and V0.5), tactile-task P300 amplitudes were higher at both tactile impairment levels than in the corresponding T0 conditions, with the largest increase observed at V0.5_T0.2. These findings indicate that tactile-task P300 responses varied systematically across degradation conditions. However, because the present study did not include a standardized behavioral measure sufficient to establish true compensation at the behavioral level, these neural differences should be interpreted as condition-dependent modulation rather than definitive proof of behavioral compensation.

### 3.6. Cross-Modal Comparisons in Visual and Tactile Tasks

To directly compare visual-task and tactile-task P300 amplitudes within each condition, we performed paired-sample *t*-tests on subject-level P300 amplitudes measured at Pz for the visual task and Cz for the tactile task. As described in the Methods, the paired differences were quantified using t values, Cohen’s d, and 95% confidence intervals, and the resulting *p* values were adjusted across the nine within-condition comparisons using the Benjamini–Hochberg false discovery rate (BH-FDR) procedure. Table 4 therefore reports both the exact *p* values and the BH-FDR-adjusted *p* values.

After BH-FDR correction, seven of the nine within-condition comparisons remained significant, whereas V0_T0.2 and V0.5_T0.5 did not survive correction. The baseline condition V0_T0 showed a significant tactile-dominant pattern, with tactile-task P300 amplitudes exceeding visual-task amplitudes. A similar tactile-dominant difference was also retained under V0_T0.5 and V0.5_T0. In contrast, V0.3_T0 and V0.3_T0.5 showed significant visual-dominant patterns, whereas V0.3_T0.2 and V0.5_T0.2 showed significant tactile-dominant patterns after BH-FDR correction. These results indicate that the relative balance between visual and tactile processing varied systematically across degradation combinations rather than remaining fixed across conditions.

Among the significant contrasts, the largest visual-dominant effect was observed under V0.3_T0.5 (Cohen’s d = 2.00), whereas the strongest tactile-dominant effect was observed under V0.5_T0.2 (Cohen’s d = −2.77). By contrast, the non-significant result at V0.5_T0.5 suggests that under severe dual-modal degradation, the difference between visual-task and tactile-task P300 amplitudes was attenuated and was no longer supported after BH-FDR correction. Likewise, the non-significant V0_T0.2 comparison indicates that under intact vision with only mild tactile degradation, no reliable within-condition task dominance was observed. Accordingly, the interpretation of modal dominance in the present study is based on the BH-FDR-corrected results reported in Table 4 rather than on uncorrected pairwise comparisons.

### 3.7. Three-Way Analysis of Variance

To further examine the joint effects of visual degradation level, tactile degradation level, and task modality on P300 amplitude, a 3 (visual: 0, 0.3, 0.5) × 3 (tactile: 0, 0.2, 0.5) × 2 (modality: visual task, tactile task) repeated-measures ANOVA was conducted on participant-level P300 peak amplitudes. For within-subject effects with more than two levels, sphericity was evaluated using Mauchly’s test, and Greenhouse–Geisser-corrected degrees of freedom and *p* values were reported when the sphericity assumption was violated. Mauchly’s test indicated that the assumption of sphericity was not violated for the main effect of visual impairment (W = 0.969, χ^2^(2) = 0.567, *p* = 0.753), the main effect of tactile impairment (W = 0.902, χ^2^(2) = 1.866, *p* = 0.393), or the tactile × modality interaction (W = 0.869, χ^2^(2) = 2.523, *p* = 0.283). However, sphericity was violated for the visual × tactile interaction (W = 0.101, χ^2^(9) = 40.007, *p* < 0.001, εGG = 0.620), the visual × modality interaction (W = 0.702, χ^2^(2) = 6.357, *p* = 0.042, εGG = 0.771), and the visual × tactile × modality interaction (W = 0.324, χ^2^(9) = 19.602, *p* = 0.021, εGG = 0.714). Therefore, Greenhouse–Geisser-corrected results are reported for these effects. A full summary of the sphericity diagnostics and the corresponding Greenhouse–Geisser-corrected ANOVA results is provided in Table 5.

The repeated-measures ANOVA revealed significant main effects of visual impairment, F(2, 38) = 47.184, *p* < 0.001, η_p_^2^ = 0.713, tactile impairment, F(2, 38) = 56.907, *p* < 0.001, η_p_^2^ = 0.750, and modality, F(1, 19) = 7.609, *p* = 0.013, η_p_^2^ = 0.286. Regarding interaction effects, the visual × tactile interaction remained significant after Greenhouse–Geisser correction, F(2.479, 47.106) = 11.705, *p* < 0.001, η_p_^2^ = 0.381, whereas the visual × modality interaction was not significant after correction, F(1.541, 29.286) = 0.829, *p* = 0.419, η_p_^2^ = 0.042. The tactile × modality interaction was significant, F(2, 38) = 4.138, *p* = 0.024, η_p_^2^ = 0.179. In addition, the three-way interaction among visual impairment, tactile impairment, and modality was significant after Greenhouse–Geisser correction, F(2.856, 54.255) = 7.791, *p* < 0.001, η_p_^2^ = 0.291. These results indicate that P300 amplitude was influenced not only by the independent effects of visual degradation, tactile degradation, and task modality, but also by the combined configuration of visual and tactile degradation and its coupling with modality, with the most robust interaction patterns observed at the visual × tactile and visual × tactile × modality levels.

## 4. Discussion

These findings are consistent with the possibility that cross-modal compensation-like neural responses vary as a function of relative sensory reliability rather than following a fixed dominance hierarchy [39,40]. However, the present EEG results do not directly demonstrate a specific neural allocation mechanism. Instead, they indicate that graded visual–tactile degradation was associated with systematic changes in oscillatory activity and P300 amplitude, and that these changes followed a condition-dependent, partly nonlinear pattern.

In particular, the P300 results showed that the largest amplitudes were observed under some moderate degradation combinations, whereas responses were attenuated under severe dual-modal degradation. This non-monotonic pattern is broadly consistent with the idea that multisensory processing efficiency may vary across levels of sensory uncertainty [41]. However, the present data do not establish a single optimal regime or demonstrate that a specific compensatory mechanism breaks down under high load. Rather, they show that neural responses differed across degradation combinations in a manner that was not simply linear. Likewise, the inverse or dissociable patterns observed between visual-task and tactile-task P300 amplitudes suggest that the relative balance between modalities changed across conditions. These observations are compatible with theoretical accounts of condition-dependent sensory weighting and cross-modal competition [42], but they should be interpreted as indirect electrophysiological evidence rather than direct proof of precision-weighted sensory reallocation. Because the present study did not include standardized behavioral performance measures sufficient to test true compensation at the behavioral level, the current findings are best understood as neural correlates consistent with compensation-like modulation under graded visual–tactile degradation. In addition, the present study did not include an independent pilot validation dataset or formal psychophysical calibration to quantify the exact reliability change induced by each manipulation level. Therefore, the visual and tactile manipulations should be interpreted as graded experimental proxies of relative reliability rather than as direct quantitative measurements of sensory signal-to-noise ratio.

## 5. Conclusions

Within a framework of progressive dual-modal (visual and tactile) degradation, this study examined the neurophysiological characteristics of cross-modal response changes under graded visual–tactile impairment. The results suggest that when one modality was degraded, neural responses associated with the other modality could increase under some conditions, as reflected in theta, alpha, and beta power modulation as well as P300 amplitude differences. The compensatory pattern was not linear, but appeared strongest under some intermediate degradation combinations and weaker under severe dual-modal degradation. In addition, the relative balance between visual-task and tactile-task responses varied across conditions, suggesting condition-dependent changes in modality weighting.

It is important to note that there are some limitations. Firstly, this study only included 20 young and healthy participants. Although the repeated measurement design improved the internal consistency at the individual level across nine visual–tactile conditions, the small sample size may still limit the stability of certain condition-specific effects and reduce the general applicability of the research results to a broader population. The sequence of the experimental blocks was randomly assigned to each participant, but it was not completely balanced; residual sequence-related effects such as learning or fatigue could not be completely excluded. Therefore, the results of this study should be understood as preliminary evidence of condition-dependent visual–tactile adaptation, rather than definitive evidence of a specific sensory reallocation mechanism. Secondly, the conclusions of this study are mainly based on the observed electroencephalogram time–frequency modulation and P300 differences under controlled laboratory conditions. To complement these neurological findings, we conducted a brief post-experiment explanation session after the experiment, and participants generally reported that different combinations of visual and tactile degradation conditions would lead to differences in their perception experience, the clarity of visual information, the usefulness of tactile cues, and the difficulty of the entire task. However, these reports are qualitative, exploratory in nature, and not collected as standardized behavioral outcome indicators. Future research should replicate this experimental paradigm in a larger and more diverse sample and include formal behavioral indicators, such as difficulty rating or recognition accuracy, in order to better evaluate the robustness and functional significance of the observed cross-modal effects.

## Figures and Tables

**Table 1:** P300 amplitude under all experimental conditions (μV, Mean ± SEM).

Visual Impairment	Tactile Impairment 0	Tactile Impairment 0.2	Tactile Impairment 0.5
0	Visual task (Pz): 3.12 ± 0.82	Visual task (Pz): 6.00 ± 0.40	Visual task (Pz): 3.55 ± 0.61
	Tactile task (Cz): 5.90 ± 0.72	Tactile task (Cz): 5.95 ± 0.74	Tactile task (Cz): 5.97 ± 0.66
0.3	Visual task (Pz): 8.60 ± 2.08	Visual task (Pz): 4.44 ± 0.53	Visual task (Pz): 10.19 ± 0.77
	Tactile task (Cz): 4.83 ± 0.62	Tactile task (Cz): 10.19 ± 1.21	Tactile task (Cz): 8.79 ± 0.60
0.5	Visual task (Pz): 2.47 ± 0.62	Visual task (Pz): 10.93 ± 1.02	Visual task (Pz): 8.01 ± 0.92
	Tactile task (Cz): 4.74 ± 0.77	Tactile task (Cz): 13.48 ± 0.81	Tactile task (Cz): 8.26 ± 0.89

**Table 2:** Cross-modal compensation percentage effects for the visual task P300 (absolute increments and relative percentages).

Visual Impairment	Comparison Conditions (Tactile Impairment)	Compensation Percentage (Visual Task)	Absolute Increment (μV)
0.3	0 → 0.2	−48.4%	−4.16
	0 → 0.5	+18.5%	+1.59
0.5	0 → 0.2	+342.5%	+8.46
	0 → 0.5	+224.3%	+5.54

Note: Take “0 → 0.2” as an example. In this statement, the “→” symbol indicates the change in the degree of tactile impairment.

**Table 3:** Cross-modal compensation percentage effects of the P300 in the tactile task (absolute increments and relative percentages).

Visual Impairment	Comparison Conditions (Tactile Impairment)	Compensation Percentage (Tactile Task)	Absolute Increment (μV)
0	0 → 0.2	+0.8%	+0.05
	0 → 0.5	+1.2%	+0.07
0.3	0 → 0.2	+111.0%	+5.36
	0 → 0.5	+82.0%	+3.96
0.5	0 → 0.2	+184.4%	+8.74
	0 → 0.5	+74.3%	+3.52

Note: Take “0 → 0.2” as an example. In this statement, the “→” symbol indicates the change in the degree of tactile impairment.

**Table 4:** Comparison of P300 amplitudes between the visual task (Pz) and the tactile task (Cz) under the nine experimental conditions.

Experiment Condition	t-Value	Exact *p* Value	BH-FDR Adjusted *p*	Cohen’s d	95% CI	Significance
V0_T0	−3.579	0.002002	0.003003	−0.82	[−4.37, −1.19]	**
V0_T0.2	0.083	0.934720	0.934720	0.02	[−1.20, 1.30]	ns
V0_T0.5	−4.784	0.000129	0.000290	−1.10	[−3.46, −1.38]	***
V0.3_T0	5.138	0.000058	0.000174	1.15	[2.28, 5.26]	***
V0.3_T0.2	−4.236	0.000447	0.000805	−0.97	[−8.52, −2.98]	***
V0.3_T0.5	8.920	0.000000032	0.000000203	2.00	[1.09, 1.71]	***
V0.5_T0	−3.120	0.005638	0.007249	−0.70	[−3.75, −0.79]	**
V0.5_T0.2	−8.730	0.000000045	0.000000203	−2.77	[−3.14, −1.96]	***
V0.5_T0.5	−0.181	0.858284	0.934720	−0.04	[−3.11, 2.60]	ns

Note: Exact *p* values were computed from the paired-sample t statistics with df = 19. Multiplicity across the nine within-condition paired comparisons was controlled using the Benjamini–Hochberg false discovery rate (BH-FDR) procedure. Significance symbols are based on the BH-FDR-adjusted *p* values: *** *p* < 0.001, ** *p* < 0.01, ns = not significant.

**Table 5:** Mauchly’s test of sphericity and Greenhouse–Geisser-corrected repeated-measures ANOVA results for P300 peak amplitude.

Effect	Mauchly’s W	χ^2^ (df)	*p* (Mauchly)	εGG	F(df1, df2)	*p* (ANOVA)	Partial η^2^
Visual	0.969	0.567 (2)	0.753	0.970	F(2, 38) = 47.184	<0.001	0.713
Tactile	0.902	1.866 (2)	0.393	0.910	F(2, 38) = 56.907	<0.001	0.750
Modality	-	-	-	-	F(1, 19) = 7.609	0.013	0.286
Visual × Tactile	0.101	40.007 (9)	<0.001	0.620	F(2.479, 47.106) = 11.705	<0.001	0.381
Visual × Modality	0.702	6.357 (2)	0.042	0.771	F(1.541, 29.286) = 0.829	0.419	0.042
Tactile × Modality	0.869	2.523 (2)	0.283	0.884	F(2, 38) = 4.138	0.024	0.179
Visual × Tactile × Modality	0.324	19.602 (9)	0.021	0.714	F(2.856, 54.255) = 7.791	<0.001	0.291

Note: For within-subject effects with more than two levels, sphericity was assessed using Mauchly’s test. When the assumption of sphericity was violated, Greenhouse–Geisser-corrected degrees of freedom and *p* values were reported. The modality factor had only two levels and therefore did not require a sphericity test. Partial eta squared (η_p_^2^) is reported as the effect size.

## Data Availability

The original contributions presented in this study are included in the article. Further inquiries can be directed to the corresponding author.
